# Aflatoxigenic *Aspergillus* Section *Flavi* Strains and Diverse Molds Isolated from California Almonds

**DOI:** 10.3390/toxins17110539

**Published:** 2025-10-31

**Authors:** Barbara Szonyi, Guangwei Huang, Tim Birmingham, Dawit Gizachew

**Affiliations:** 1 Department of Chemistry and Physics, Purdue University Northwest, Hammond, IN 46323, USA; 2 Almond Board of California, 1150 Ninth St., Ste. 1500, Modesto, CA 95354, USA

**Keywords:** almonds, *Aspergillus*, *Aspergillus flavus*, aflatoxins, fungus, molds, mycobiota, mycotoxins, nuts

## Abstract

Almonds are an essential crop for the economy of California. However, mold and mycotoxin contamination of this commodity has a serious impact on food safety and international trade. The contamination levels of molds and the aflatoxigenic potential of *Aspergillus* section *Flavi* isolates were studied on almonds collected at a processing plant in California. The mean total fungal count for 80 samples was 1.0 × 10^4^ CFU/g, while 62 samples (77.5%) had a total mold count less than 1.0 × 10^4^ CFU/g. The most common fungal contaminants were *Aspergillus* section *Nigri* (100% of samples), followed by *Penicillium* (57.5%) and *Cladosporium* (52.5%) species. *Rhizopus*, *Fusarium* and *Alternaria* spp. were less frequent. A total of 26 *A*. section *Flavi* strains were identified, with most strains (23) belonging to the L morphotype of *A. flavus*. In addition, two S morphotypes of *A. flavus*, and one *A. tamarii* strain were observed. Other *Aspergillus* species, including *A. terreus* and *A. ochraceus* were rare. High Performance Liquid Chromatography (HPLC) analysis revealed that 9 out of 13 isolated *A. flavus* strains produced aflatoxin B_1_ (AFB_1_) on yeast extract sucrose media. The highest levels of AFB_1_ were produced by two *A. flavus* isolates belonging to the S morphotype (78 and 260 µg/kg). Increasing temperatures and drought conditions may change the population dynamics of toxigenic mold strains on almonds, emphasizing the need to continue monitoring these fungal populations.

## 1. Introduction

Almond (*Prunus amygdalus*) is the most important commercial tree nut in the world with a global annual production of 3.5 billion pounds (1.6 billion kg). The United States is the largest producer of almonds, with the state of California producing 76% of the total global almond output in the crop year 2023/2024 [1]. Almonds are also the most valuable specialty crop export in the USA, with a production value exceeding $4.6 billion per year [1]. Nevertheless, almonds are susceptible to contamination with molds that may produce toxic secondary metabolites known as mycotoxins [2]. Mycotoxin contamination of crops is a serious food safety issue, due to severe adverse effects on human and animal health. Almonds are exposed to airborne or soil-borne fungal spores in the field or during harvest and processing. These spores settle and remain on the surface of the kernels. During processing, storage and shipment, almonds are maintained under low water activity that inhibits active fungal growth. However, due to the presence of fungal spores on the nuts, even a temporary increase in water activity may lead to mold growth and serious mycotoxin contamination [3]. Most of the mycotoxins which are considered important food contaminants are produced by the fungal genera *Aspergillus*, *Fusarium,* and *Penicillium* [2].

Aflatoxins are the most potent and dangerous group of mycotoxins with carcinogenic, cytotoxic, immunosuppressive and mutagenic effects. Chronic exposure to aflatoxins is a main cause of liver cancer, and the International Agency for Research on Cancer (IARC) classified several aflatoxins as highly carcinogenic for humans and animals [4]. Due to their harmful effects, numerous countries regulate aflatoxins in their domestic food supply. The United States set a limit of 20 µg/kg of total aflatoxins for domestic human food [5]. Also, the European Union (EU) has established maximum permissible levels of 10 µg/kg for total aflatoxins and 8 µg/kg for aflatoxin B_1_ for imported nuts intended for human consumption [6]. The stringent aflatoxin restriction of the EU is a concern to the California almond exporters, because exceeding the regulatory limit may result in crop rejection and significant monetary losses.

Aflatoxins are a group of mycotoxins produced by *Aspergillus* fungal species belonging to the *Flavi* section. The main aflatoxigenic species in *Aspergillus* section *Flavi* affecting almonds are *A. flavus* and to a lesser extent, *A. parasiticus* [7]. In previous studies, *A. flavus* populations have been found to be markedly diverse regarding their morphology and toxigenicity. Members of the S strain produce numerous, small sclerotia (<400 µm) and consistently high levels of B aflatoxins, while L strains have fewer, large sclerotia (>400 µm) and variable concentrations of B aflatoxins [8]. Overall, less than half of the known *A. flavus* strains produce aflatoxins [9]. Conversely, *A. parasiticus* strains are more uniform in their morphology and toxigenicity, and they produce consistently high levels of B and G type aflatoxins [7]. Another member of the section *Flavi* group, *A. tamarii*, can produce other mycotoxins such as cyclopiazonic acid (CPA). Other *Aspergillus* species, such as *A. niger* and *A. carbonarius* in the *Nigri* section, as well as *A. ochraceus,* are known to produce ochratoxins. Ochratoxin A is a carcinogenic, cytotoxic and immunosuppressive mycotoxin [10], which has regulatory limits in many commodities in several countries [11]. While Ochratoxin A (OTA) is not regulated in the United States, the European Commission set regulatory limits in food commodities [12], which makes this mycotoxin an important consideration for the international trade of almonds.

According to past research on fungal populations, almonds are highly susceptible to contamination with toxigenic molds. Several surveys of the almond mycobiota revealed high frequencies of contamination with *Aspergillus* section *Flavi* and *Nigri* members [2,3,13]. The presence of these toxigenic molds could compromise the quality of the almonds and cause serious health concerns for consumers, as well as major economic losses to the almond industry. The ongoing changes in climate conditions and pest management practices, along with stringent regulatory limits for mycotoxins, necessitate continued monitoring of the mycological quality of almonds. The aims of this study were to, firstly, identify the presence, prevalence and contamination levels of mold species and, secondly, determine the aflatoxigenic potential of isolated *Aspergillus* section *Flavi* strains in California almonds.

## 2. Results

In all, 80 almond samples from 10 lots and six different processing streams (Input, Electronic #1 (E1), Electronic #2 (E2), Electronic #3 (E3), Hand Sort, and Output) were analyzed. Two samples per lot were collected for both Input and Output streams. Only one sample per lot was collected for each of the processing streams E1, E2, E3 and Hand Sort. Thus, a total of eight samples were analyzed in each lot.

### 2.1. Total Mold Counts

**Figure 1:**
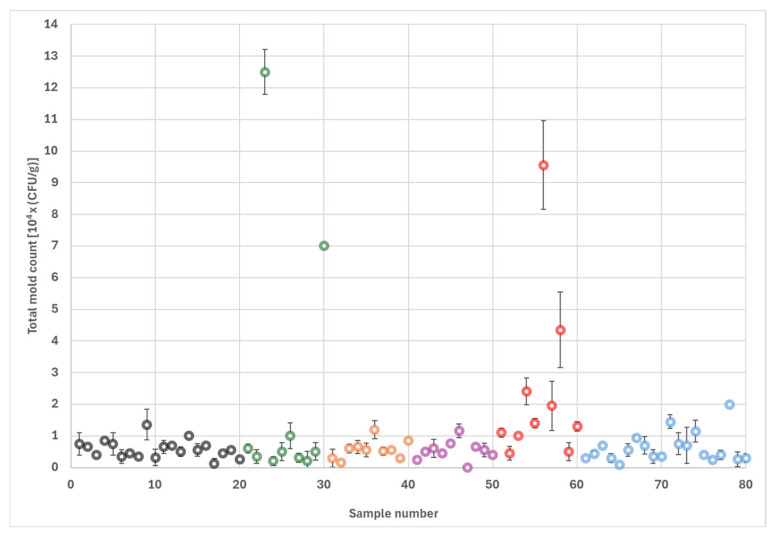
Total mold counts of 80 California almond samples showing differences in screening standards between electronic sorting and manual sorting. Different processing streams are indicated with the following colors: Input stream—Black; Electronic stream #1—Green; Electronic stream #2—Orange; Electronic stream #3—Purple; Hand Sort stream—Red; Output stream—Blue. Standard deviations of duplicate plates are also shown.

As shown in Figure 1, 62 samples (77.5%) had a total mold count less than 1.0 × 10^4^ CFU/g, while 14 samples (17.5%) contained 1.0–2.4 × 10^4^ CFU/g. The four highest total mold counts were 4.3, 7.0, 9.6 and 12.5 × 10^4^ CFU/g. The average and median total mold counts for all 80 samples were 1.0 × 10^4^ and 5.5 × 10^3^ CFU/g, respectively. There was no statistically significant difference in total mold counts among the 10 lots.

**Figure 2:**
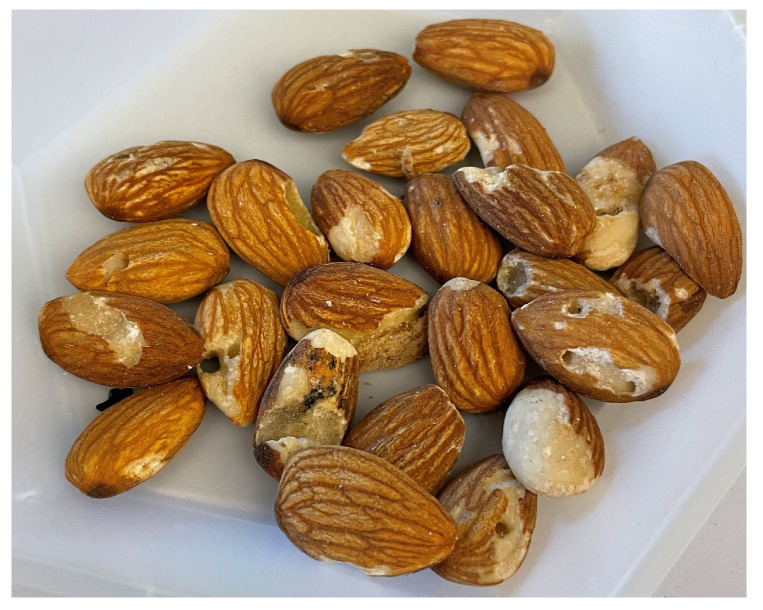
Almonds collected from the Hand Sort reject stream at a California processing plant, showing that highly damaged kernels were picked out manually during processing.

Tukey’s Honestly Significant Difference test revealed that rejects from the processing streams E1 and Hand Sort had significantly higher levels of fungal contamination compared to almonds from the other four streams (Table 1). The statistical analysis was repeated with the removal of the four highest total fungal counts as outliers. As a result, fungal counts from only Hand Sort rejects remained significantly higher than those from the other streams (Figure 2).

### 2.2. Fungal Identification and Contamination Levels

**Figure 3:**
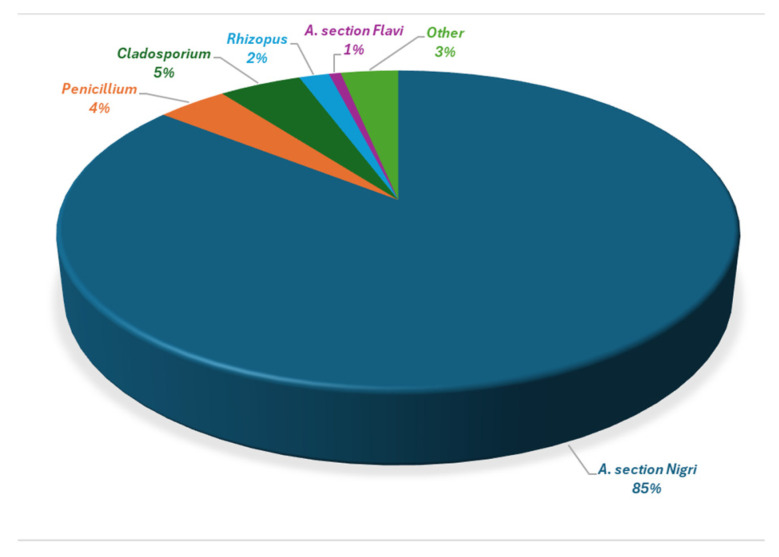
Relative contribution of different fungal groups to the total mold count in 80 California almonds samples.

Molds were identified to the genus level, while *Aspergillus* species were identified to the section or species level. According to Figure 3, 85% of the total mold count was attributed to species belonging to *Aspergillus* section *Nigri*. Only 1% of all molds that were encountered belonged to the potentially aflatoxigenic strains of *A*. section *Flavi*. *Penicillium* and *Cladosporium* species contributed 4 and 5% towards the total mold count, respectively.

Table 2 lists the genera or species of molds identified in this study and their respective contamination levels. Also listed are the percentages of samples (sample prevalence) and lots (lot prevalence) containing each group. The most common mold contaminants were *Aspergillus* section *Nigri* (100% of samples), followed by *Penicillium* (57.5%) and *Cladosporium* (52.5%) species. These molds were detected in all 10 lots. Nearly one third of all samples (31%) contained *A. flavus*. Also, *Rhizopus*, *Alternaria*, and *Fusarium* species were frequently observed. Other *Aspergillus* species (*A. ochraceus*, *A. terreus* and *A. tamarii*) were rare. The mean contamination level of *A. flavus* was 1.0 × 10^3^ CFU/g, while *A.* section *Nigri* was present at an average level of 9.4 × 10^3^ CFU/g.

### 2.3. Characterization of A. flavus Strains

**Figure 4:**
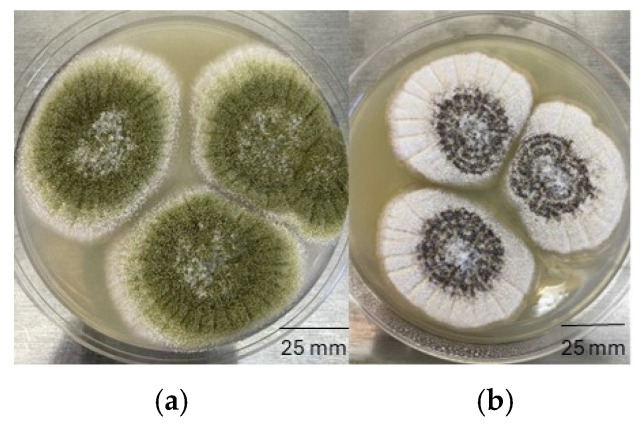
Macroscopic features of *Aspergillus flavus* strains isolated from almonds and grown on Potato Dextrose Agar at 25 °C for 7 days. (**a**) *A. flavus* L morphotype (**b**) *A. flavus* S morphotype.

Colonies with greenish colored spores were transferred to Potato Dextrose Agar (PDA) for purification and to AFPA for identification of potentially aflatoxigenic strains. A total of 25 *A. flavus* strains were detected. Twenty-three strains belonged to the L morphotype and were characterized by white, floccose mycelia and the presence of abundant green conidial heads spread uniformly over the colony (Figure 4). In contrast, the mycelia of two *A. flavus* isolates had a rough, grainy texture, with dark brown sclerotia dominating colony appearance, and suppressed conidia production. These latter features are consistent with *A. flavus* strains of the S morphotype. Microscopically, *A. flavus* strains were characterized by biseriate conidiophores with radial orientation. Vesicles were spherical, varying in size from 25 to 50 µm in diameter. Metulae covered ¾ or the entire surface of the vesicles. Conidia were spherical with approximately 4–5 µm in diameter.

### 2.4. Aflatoxin Production

Thirteen *A. flavus* strains isolated from 8 different lots were assessed for their ability to produce aflatoxins on Yeast Extract Sucrose (YES) media. Four strains produced negligible amounts of aflatoxins (<7 µg/kg total) while seven strains produced total aflatoxins in the range of 12–54 µg/kg (Table 3). The two S strains synthesized the highest levels of B_1_ as well as total aflatoxins. The production of aflatoxins B_2_, G_1_ and G_2_ were low in all 13 strains.

## 3. Discussion

The goal of this work was to evaluate the mycobiota and to determine the aflatoxigenic potential of *Aspergillus* section *Flavi* strains isolated from California almonds. To this end, 80 almond samples were collected at a large processing plant and tested for the presence and levels of molds. The water activity of the almonds reflected normal processing conditions, which is too low for active fungal growth. Therefore, the molds that were isolated in this study grew from fungal spores that colonized the surface of the almonds in the field or during harvest [3,14].

The dominant fungal contaminant was *Aspergillus* section *Nigri,* which was encountered in every sample and accounted for 85% of the total fungal count, followed by *Penicillium* and *Cladosporium* species. *Aspergillus* section *Flavi* members were also frequently observed (31% of the samples) but only constituted 1% of the total fungal load. Other *Aspergillus* species, including *A. tamarii*, *A. terreus* and *A. ochraceus*, were present at low levels.

Surveys conducted in the past revealed similar mold contamination profiles in almonds. For example, Bayman et al. [3] reported that *Penicillium*, *Aspergillus*, and *Rhizopus* spp were the most frequently encountered molds in a large study using field-collected and market-bought almonds in California. An older survey conducted on Nonpareil almonds in California showed that the most prevalent molds were in the *A.* section *Nigri* group, while *Rhizopus*, *Pencillium,* and *Cladosorium* species were also abundant [14]. The same study found *A. flavus* colonies in 30% of all samples, which is similar to the sample prevalence of *A. flavus* detected in the present study. Furthermore, Kenjo et al. [15] found *A*. sections *Nigri* and *Flavi* as well as *Penicillium*, *Cladosporium*, and *Rhizopus* spp. in 30 samples of commercial almond powder imported into Japan. In the same study, total fungal counts ranged between 10^2^–10^4^ CFU/g, which is comparable to the total mold counts encountered in the present study.

The almonds in this work were heavily contaminated with the potentially toxigenic molds belonging to *Aspergillus* section *Nigri*. Members of this section include *Aspergillus niger*, *A. carbonarius*, *A. tubingensis* and several other species [16]. Due to their morphological similarities, genetic analysis is required to reliably differentiate the species within this group. The present study relies on morphological characteristics for fungal identification, which is a valid but limited approach, especially for differentiating species within the *Aspergillus* section *Nigri* group. Molecular methods, such as sequencing of the ITS, β-tubulin, or calmodulin genes [11] are required for definitive species identification for further studies. The section *Nigri* group can produce 145 different secondary metabolites, many of which are toxic to humans and animals [2]. For example, *A. niger* can produce OTA and fumonisin B_2_ (FB_2_), while the majority of *A. carbonarius* strains produce OTA [2]. Fumonisins are another group of carcinogenic and cytotoxic mycotoxins with a maximum limit set for maize-based foods in the EU [12].

Several authors similarly found high prevalence and contamination levels of *A.* section *Nigri* members in almonds grown in California [3,14,17]. Tournas et al. [13] studied the mycological profiles of tree nuts, including 17 almond samples sold in supermarkets in the Washington DC area, and found that A. section *Nigri* species were the most common contaminants with levels up to 10^4^ CFU/g. In the present study, contamination levels of *A.* section *Nigri* reached up to 10^5^ CFU/g in some samples. Palumbo et al. [11] demonstrated that *A.* section *Nigri* members were the most frequent contaminants in inedible, pick-out California almond samples, infecting 18 out of 21 samples. These investigators recovered *A*. section *Nigri* populations at levels up to 1.2 × 10^5^ CFU/g, which is comparable to our results. Also, of the 34 *A. niger* strains isolated in the same study, 72% produced FB_2_. At the same time, none of the *A.* section *Nigri* strain produced OTA in the study by Palumbo et al. The consistent and heavy contamination levels of California almonds with the potentially toxigenic *A.* section *Nigri* members is an important consideration for both consumer exposure and the export trade of almonds. Though our current research focused on aflatoxins, further studies are recommended to investigate the OTA and fumonisin production of *A.* section *Nigri* strains in California almonds.

A total of 26 *A*. section *Flavi* strains were isolated in the present work, with most strains (23) belonging to the L morphotype of *A. flavus*. In addition, two S morphotypes of *A. flavus*, and one *A. tamarii* strain were observed. Furthermore, nine out of the 13 *A. flavus* isolates produced aflatoxins, particularly B_1_, on YES media, with the S strains generating the highest levels of aflatoxins. In a study on the population structure of *A.* section *Flavi*, the L strain of *A. flavus* was the most frequently encountered member in the soils of almond orchards in California, while the more toxigenic S strains were also present at lower frequencies [7]. In contrast, the highly toxigenic S strains are more abundant in traditionally hot and dry regions such as Arizona and Texas. Furthermore, the proportion of S strains in *A. flavus* populations increases with soil temperature [7]. Therefore, changing climatic conditions, specifically droughts and increasing temperatures, might create conditions that are favorable to the expansion of highly toxigenic S strains into areas that have been previously dominated by L strains. Due to the trend of climate warming, we recommend improving the prediction and prevention of the spread of S-type strains.

In agreement with our study, Tournas et al. [13] did not detect any *A. parasiticus* strains on almonds. Similarly, a large survey of field-collected and store-bought California almonds reported the high occurrence of *A. flavus,* while *A. parasiticus* was rarely found [3]. In a survey of Portuguese almonds, however, Rodriguez et al. [9] reported that 56% of the *A.* section *Flavi* isolates belonged to *A. parasiticus*. These results show the impact of geographic variation on the biodiversity of Section *Flavi* strains. Temperature and precipitation are also known to influence the structures of *A.* section *Flavi* communities in almond orchards in California. Warmer temperature and low precipitation are thought to result in lower rates of *A. parasiticus*. For example, when California experienced consecutive dry years from 2007 to 2009, an increase in the proportion of *A. flavus* isolates was observed [7]. As the almond growing regions experience increased temperatures and reduced precipitation, the distribution and abundance of *A.* section *Flavi* strains in California almond orchards can be expected to change. Hence, it is essential to carefully monitor the prevalence and population structure of aflatoxigenic fungi on almonds. Molecular typing of *A.* section *Flavi* strains is a useful tool to shed light on the genetic diversity of this population.

In addition to *Aspergillus* section *Nigri* and *Flavi* species, members in the genera *Penicillium* and *Cladosporium* were abundant in the present work, infecting at least half of all samples in all ten lots. *Cladosporium* spp. are major spoilage fungi in a wide range of crops, but they are not considered significant mycotoxin producers [18]. On the other hand, *Penicillium*, along with *Aspergillus*, are dominant representatives of storage fungi, capable of producing a wide range of mycotoxins. Some members of the *Penicillium* genus can produce mycotoxins such as citrinin, OTA, penicillic acid, and CPA [2]. While prior surveys consistently reported *Pencillium* spp as a frequent contaminant in tree nuts, including almonds [3,13,18], to date, very few studies have been devoted to determine the mycotoxigenic potential of these mold species.

In the present work, almonds were collected at a large processing plant. After harvest, almonds are shipped to processing plants, where shells and debris are removed and the whole almonds are stored in large storage units named lots. Each lot is subjected to a processing system designed to methodically remove kernels with defects. During processing, the acceptable almonds are separated from those with defects such as mechanical or insect damage [19]. The almonds in this study included rejects from electronic sorting lines and from a hand sorting line. The results indicated that the hand sort reject stream had significantly higher levels of mold contamination compared to the other processing streams. This finding may be due to differences in screening standards between electronic and manual sorting. Also, the positive correlation between kernel damage and fungal invasion in almonds has been well documented [3,8]. It is therefore possible that hand sorting removes the most damaged kernels with the highest levels of fungal contaminants.

## 4. Conclusions

In conclusion, this study reports high levels of contamination with potentially toxigenic *Aspergillus* section *Nigri* strains as well as a high proportion of aflatoxigenic section *Flavi* strains in almonds from a large processing plant in California. While current storage and processing conditions inhibit the growth of molds, the presence of toxic mold spores on the almond kernels may constitute a food safety hazard if the water activity is not effectively controlled. Increasing temperatures and drought conditions may change the population dynamics of toxigenic mold strains on almonds, necessitating ongoing monitoring of these fungal populations.

## 5. Materials and Methods

### 5.1. Sample Collection

Whole almond kernels of the Nonpareil variety were collected at a large almond processing plant in Northern California. The plant receives almonds from growers located in the Sacramento River Valley (39° N, 121° W). This region is characterized by hot and dry summers, and mild to cool and wet winters. The processing plant receives almonds from several orchards and pools nuts in lots. Lots are large, enclosed storage units that contain 42,000–44,000 pounds (19,000–20,000 kg) of almonds each. Eight composite samples were collected from each of 10 different lots at various stages of the sorting process following a stratified random sampling design. For all processing streams, the almonds were thoroughly mixed and 44 increments of 1 kg each were randomly collected. All increments were subsequently thoroughly mixed, and a composite sample of 50 g was randomly collected. For each lot, two composite samples were collected from both the Input and Output processing streams. In addition, one composite sample per lot was collected at each of the following reject streams: Electronic #1 (E1), Electronic #2 (E2), Electronic #3 (E3) and Hand Sort. Almonds collected from reject streams were sorted out and considered unfit for human consumption (also called pick-outs). The samples were stored at 4 °C in plastic bags until analysis, because it has been shown that there are no significant changes in fungal populations of almonds stored at this temperature [14]. The water activity of the almonds was 0.56 a_w_, which prevented fungal growth during storage. The water activity was measured using a water activity meter (HygroPalm23Aw, Rotronic, Bassersdorf, Switzerland).

### 5.2. Mold Enumeration

Mold count was determined using Dichloran 18% Glycerol (DG18) agar (Millipore Sigma, Burlington, MA, USA), which is suitable for the enumeration of xerophilic molds from dried foods as outlined in ISO 21527-2:2008 [20]. This media contains Dichloran, which controls the growth of rapidly spreading molds such as *Rhizopus*, allowing the identification of slower growing fungi. An initial suspension was prepared by mixing 25 g of almond samples and 225 mL of sterile 0.1% peptone water (Millipore Sigma, Burlington, MA, USA) with 0.05% Tween 80 in a sterilized Waring blender (Waring Laboratory, Torrington, CT, USA) for 45 s on the high setting. Further ten-fold dilutions were obtained by mixing 1 mL of suspension with 9 mL of sterile peptone water to obtain a decimal dilution series. Immediately after blending, 0.1 mL of aliquots of each dilution were plated in duplicates on standard 100 × 15 mm sterile Petri dishes (Corning, NY, USA). The inoculum was spread evenly with a sterile L-shaped cell spreader (Termo Fisher Scientific, Waltham, MA, USA) using the spread plate technique. The inoculated DG18 plates were incubated at 25 °C for 5 days. After incubation, the colonies were counted, and the mean counts of the duplicate plates were reported as colony-forming units per gram (CFU/g).

### 5.3. Identification of Molds and A. Section Flavi Strains

After determining the mold count on DG18 plates, colonies of representative molds were selected and cultivated on Potato Dextrose Agar (PDA) and Czapek-Dox Agar (Millipore Sigma, Burlington, MA, USA) at 25 °C for 7 days. The following macroscopic features were observed for the isolates: colony size, color and texture; reverse color of the colony; color of mycelia and spores; presence of exudate or sclerotia. After assessing the macroscopic features of the isolates, wet mounts were prepared for examination with a bright field microscope (Termo Fisher Scientific, Waltham, MA, USA). Lacto-fuchsin stain and immersion oil (Millipore Sigma, Burlington, MA, USA) were used as needed to assist with visualization. Microscopic features included the size, color and shape of spores, fruiting structures and hyphae, and the presence of cleistothecium/ascospores. *Aspergillus* species were further evaluated based on size and morphology of the conidiophores and conidia. Isolates were identified to genus or species level using keys described by Pitt and Hocking [21]. Potentially aflatoxigenic *Aspergillus* isolates were identified and cultured on *Aspergillus Flavus* and *Parasiticus Agar* (AFPA) plates (Millipore Sigma, Burlington, MA, USA) and incubated at 30 °C. Colonies of *A. flavus* and *A. parasiticus* were distinguished by bright orange-yellow reverse colors after 48 h. At the same time, *Aspergillus tamarii*, another member of the *A.* section *Flavi* group, was identified by a dark brown reverse colony color on AFPA under the same incubation conditions [21].

### 5.4. Determination of Aflatoxigenic Potential of A. Section Flavi Isolates

Spore inoculum was prepared from isolated, pure *A. flavus* cultures grown on Potato Dextrose Agar (PDA) at 25 °C for seven days. Spores were aseptically harvested and suspended in sterile 0.01% Tween 80 solution (MP Biomedicals, Solon, OH, USA). The spore suspension was adjusted to contain 10^6^ spores/mL as determined by a hemocytometer (INCYTO, Chungnam-do, Republic of Korea). Sterile culture tubes of Yeast Extract Sucrose (YES) broth were inoculated with 1 mL of spore suspension. The YES broth was prepared by dissolving 22 g of yeast extract (Millipore Sigma, Burlington, MA, USA) and 180 g of sucrose in 1000 mL of DI water, with a final pH of 6.5. The tubes were incubated as stationary cultures at 27 °C for 14 days. After the incubation period, 15 mL of methanol was added to the tubes, and the contents were rigorously homogenized. The mycelium was separated by PF Filter Paper (Thermo Fisher Scientific, Waltham, MA, USA) and the filtered extract was collected. Next, 5 mL of the filtrate was mixed with 5 mL of deionized (DI) water, and the diluted extract was filtered again using a 25 mm syringe filter (Thermo Fisher Scientific, Waltham, MA, USA). The filtrate was transferred to immunoaffinity columns (Vicam, Milford, MA, USA), washed twice with DI water, and the aflatoxin was eluted at a rate of 1 drop/s with 1ml of HPLC-grade methanol.

Aflatoxin analysis was carried out using high performance liquid chromatography (Thermo Scientific Ultimate 3000 HPLC) with fluorescence detector at 365 nm excitation and 455 nm emission. Aflatoxins were analyzed with isocratic mobile phase (10% acetonitrile, 40% methanol and 50% water) and a stationary reverse phase column (C18, 4.6 mm × 250 mm). Linear calibration curves were prepared for AFB_1_, AFB_2_, AFG_1_ and AFG_2_ separately using a mixture of nine standard aflatoxin solutions (Restek, Bellefonte, PA, USA). The calibration curves for AFB_1_, AFB_2_, AFG_1_ and AFG_2_ were linear with r^2^ = 0.993, 0.9979, 0.996 and 0.9991, respectively. The detection and quantitation limits were 1.5 and 2.0 µg/kg, respectively, under the conditions described above.

### 5.5. Statistical Analyses

Means and standard deviations were calculated for duplicate plates for each of the 80 samples. Outliers were defined as observations that deviated at least three times the standard deviations (SD) from the means. For each type of fungus, the sample prevalence was calculated as the number of samples that contained the fungus, divided by 80 (the total number of samples). Similarly, lot prevalence was obtained by dividing the number of lots containing the fungus by 10 (the total number of lots). Both sample and lot prevalences were multiplied by 100 to obtain percentages. The statistical difference in total mold counts among different sorting streams and lots was investigated using Tukey’s Honestly Significant Difference test with standard statistical software (STATA version IC15, College Station, TX, USA). A *p*-value of equal or less than 0.05 was considered significant.

## Figures and Tables

**Table 1:** Total mold counts of almonds collected at different processing streams in California.

Processing Stream		All Samples (*n* = 80)					Outliers Removed (*n* = 76)		
	*n*	Mean (CFU/g)	SD	Tukey’s HSD		*n*	Mean (CFU/g)	SD	Tukey’s HSD
Input	20	5.8 × 10^3^	6.7 × 10^2^	a		20	5.8 × 10^3^	6.7 × 10^2^	a
Electronic 1	10	2.3 × 10^4^	4.3 × 10^3^	b		8	4.9 × 10^3^	9.9 × 10^2^	a
Electronic 2	10	5.6 × 10^3^	9.2 × 10^2^	a		10	5.6 × 10^3^	9.2 × 10^2^	a
Electronic 3	10	6.2 × 10^3^	9.4 × 10^2^	a		10	6.2 × 10^3^	9.4 × 10^2^	a
Hand Sort	10	2.4 × 10^4^	4.4 × 10^3^	b		8	1.3 × 10^4^	1.0 × 10^3^	b
Output	20	6.2 × 10^3^	6.8 × 10^2^	a		20	6.2 × 10^3^	6.8 × 10^2^	a

Note: *n* denotes sample size. SD: Standard deviation. Tukey’s HSD: Tukey’s Honestly Significant Difference test statistics. Groups that have different letters are significantly different from each other.

**Table 2:** Frequencies and contamination levels of fungal groups identified in 80 California almond samples from 10 different lots.

Identity	Number (%) of Contaminated Samples (*n* = 80)	Number (%) of Contaminated Lots (*n* = 10)	Mean of Positive Samples (CFU/g)	Range of Count (CFU/g)
*Alternaria* spp	8 (10)	4 (40)	1.7 × 10^3^	<10^2^ to 5.0 × 10^3^
*A. ochraceus*	5 (6.3)	3 (30)	2.0 × 10^3^	<10^2^ to 3 × 10^3^
*A. flavus*	25 (31.2)	8 (80)	1.0 × 10^3^	<10^2^ to 10^3^
*A.* section *Nigri*	80 (100)	10 (100)	9.4 × 10^3^	3.0 × 10^2^ to 1.3 × 10^5^
*A. tamarii*	1 (1.3)	1 (10)	1.0 × 10^3^	<10^2^ to 10^3^
*A. terreus*	3 (3.7)	2 (20)	1.0 × 10^3^	<10^2^ to 10^3^
*Cladosporium* spp.	42 (52.5)	10 (100)	1.7 × 10^3^	<10^2^ to 2.0 × 10^4^
*Fusarium* spp	17 (21.3)	7 (70)	1.7 × 10^3^	<10^2^ to 5.0 × 10^3^
*Mucor* spp	1 (1.3)	1 (10)	1.0 × 10^3^	<10^2^ to 10^3^
*Paecylomyces* spp	5 (6.3)	3 (30)	1.0 × 10^3^	<10^2^ to 10^3^
*Penicillium* spp.	46 (57.5)	10 (100)	1.3 × 10^3^	<10^2^ to 2.0 × 10^4^
*Rhizopus* spp.	28 (35)	8 (80)	1.0 × 10^3^	<10^2^ to 10^4^

Note: *n* denotes sample size.

**Table 3:** Production of Aflatoxins B_1_, B_2,_ G_1_ and G_2_ and total aflatoxins (B_1_ + B_2_ + G_1_ + G_2_) on Yeast Extract Sucrose media by *Aspergillus flavus* strains isolated from California almonds (µg/kg). The sample ID reflects the lot number and the processing stream where the strain was isolated from.

Sample ID	B_1_	B_2_	G_1_	G_2_	Total
12-E2	17	ND	8	ND	25
13-E1	12	ND	ND	ND	12
13-H *	78	4	3	ND	85
14-IN	52	2	ND	ND	54
14-E1 *	260	ND	ND	ND	260
15-E3	33	2	6	ND	41
15-OUT	2	ND	ND	ND	2
17-IN	14	ND	ND	ND	14
17-OUT	ND	ND	ND	ND	ND
18-E3	12	ND	ND	ND	12
19-E1	ND	ND	4	ND	4
19-OUT	26	ND	ND	ND	26
20-H	7	ND	ND	ND	7

* Denotes S morphotype of *A. flavus*. ND: Not detected.

## Data Availability

The original contributions presented in the study are included in the article; further inquiries can be directed to the corresponding author.

## Abbreviations

The following abbreviations are used in this manuscript:

AFB	Aflatoxin B
AFG	Aflatoxin G
CPA	Cyclopiazonic acid
OTA	Ochratoxin A
